# Die Diamorphinbehandlung Opioidabhängiger – zur Novellierung der Indikationskriterien nach Betäubungsmittel-Verschreibungsverordnung

**DOI:** 10.1007/s00115-025-01888-6

**Published:** 2025-09-12

**Authors:** N. Scherbaum, M. Specka, N. Wodarz, U. Bonnet, P. Roser

**Affiliations:** 1 LVR-Universitätsklinik Essen, Klinik für Psychiatrie und Psychotherapie sowie Klinik für Abhängiges Verhalten und Suchtmedizin, Medizinische Fakultät der Universität Duisburg-Essen, Virchowstr. 174, 45147 Essen, Deutschland; 2 Zentrum für Suchtmedizin, Klinik und Poliklinik für Psychiatrie und Psychotherapie der Universität am Bezirksklinikum Regensburg, Regensburg, Deutschland; 3 Klinik für seelische Gesundheit, EVK Castrop-Rauxel, Castrop-Rauxel, Deutschland; 4 Klinik für Psychiatrie, Psychotherapie und Psychosomatik, Zentrum für Abhängigkeitserkrankungen, Psychiatrische Universitätsklinik Zürich, Zürich, Schweiz

## Einleitung

Seit 2009 kann Diamorphin (eigentlich Diacetyl-Morphin; chemischer Name für Heroin) in der Substitutionsbehandlung Opioidabhängiger zulasten der gesetzlichen Krankenkassen verschrieben werden. Vorangegangen war u. a. eine in Deutschland durchgeführte Studie, die bei Heroinabhängigen mit vorrangig intravenösem Konsum die Überlegenheit einer Kombinationsbehandlung aus intravenös appliziertem Diamorphin und Methadon gegenüber einer alleinigen Methadonbehandlung in Hinblick auf die Reduktion des Konsums illegal erworbenen Heroins und die Haltequote in Behandlung belegte [6]. Untersuchungen in anderen Ländern kamen zu ähnlichen Ergebnissen [4, 5, 10]. Risiken der Diamorphinbehandlung sind insbesondere die Diamorphinintoxikation mit Atemdepression sowie epileptische Krampfanfälle. Praxen bzw. Ambulanzen müssen daher eine Beobachtungszeit nach Einnahme einplanen sowie auch Erste-Hilfe-Maßnahmen vorhalten. Bei einer Halbwertszeit von etwa 6 h wird Diamorphin mehrfach täglich appliziert im Vergleich zu der einmal täglichen Einnahme lang wirksamer Opioide wie Methadon und Buprenorphin. Dies bedeutet längere Öffnungszeiten (und mehr Personal) für die Praxen.

In der Substitutionsbehandlung mit lang wirksamen Opioiden gelingt der Mehrheit der Betroffenen die Reduktion des Konsums illegalen Heroins und die Verbesserung der körperlichen Gesundheit (für Deutschland: [8]). Eine Minderheit, z. T. Schwerstabhängige genannt (international auch „refractory heroin addiction“; [10]), profitiert jedoch nicht und bricht die Therapie oft ab. Bei Unterschieden im Detail betrafen die Einschlusskriterien der kontrollierten Untersuchungen zu Diamorphin intravenös konsumierende Heroinabhängige, die von vorangegangenen konventionellen Substitutionsbehandlungen nicht profitiert hatten. Für diese Gruppe ist die Diamorphinbehandlung eine gut evaluierte Therapieoption.

## Rechtliche Regeln der Substitutionsbehandlung in Deutschland

Die Substitutionsbehandlung unterliegt in Deutschland insbesondere der Betäubungsmittel-Verschreibungsverordnung (BtMVV) sowie der Richtlinie der Bundesärztekammer (BÄK-RL). In der BtMVV (§ 5a; 2009) sowie der BÄK-RL (2010) wurden die Indikationskriterien für die Diamorphinbehandlung nach den Einschlusskriterien der in Deutschland durchgeführten Untersuchung definiert: Mindestalter 23 Jahre, mindestens 5‑jährige Heroinabhängigkeit, vorrangig intravenöser Konsum, zwei erfolglos beendete Behandlungen der Abhängigkeit, davon mindestens eine mindestens 6‑monatige Substitutionsbehandlung. Die BÄK-RL betont zudem, dass die Diamorphinbehandlung „bei schweren Verläufen“ eingesetzt werde (§ 2).

Im Februar 2025 wurden die Indikationskriterien für die Diamorphinbehandlung in der BtMVV geändert. Diese lauten jetzt folgendermaßen (siehe im Vergleich Tab. 1): Mindestalter 18 Jahre, mindestens zweijährige Opioidabhängigkeit, erhebliche Defizite in medizinischer, psychologischer oder sozialer Hinsicht, die jeweils auf den Missbrauch von unerlaubt erworbenen oder erlangten Opioiden zurückzuführen sind, insgesamt mindestens sechsmonatige Substitutionsbehandlungen, die sich als nicht geeignet oder als erfolglos erwiesen haben. Bei Patienten im Alter zwischen 18 und 23 Jahren muss eine Zweitmeinung bei einem suchtmedizinisch qualifizierten Arzt zur Indikationsstellung eingeholt werden, der nicht der gleichen Institution angehört.

**Tab. 1:** Kriterien für Diamorphinbehandlung nach BtMVV § 5a

	Alte Version	Neue Version
Mindestalter	23 Jahre	18 Jahre*
Dauer der Opiatabhängigkeit	5 Jahre	2 Jahre
Applikationsform	Vorwiegend intravenös	Keine Angabe
Vorhergehende Behandlungen	*Zwei* erfolglose Behandlungen, davon eine mindestens 6‑monatige Substitutionsbehandlung	Erfolglose Substitutionsbehandlungen von insgesamt mindestens 6‑monatiger Dauer
Fallschwere	Schwerwiegende körperliche und psychische Funktionsstörung	Erhebliche medizinische, psychologische oder soziale Defizite

*Bei Personen im Alter von 18 bis 23 Jahren ist für die Indikationsstellung eine Zweitmeinung erforderlich

## Diskussion

Die Vielfalt der verfügbaren Substitutionsmittel einschließlich des Diamorphins ist therapeutisch wertvoll. Zum 01.07.2024 befanden sich 1,8 % der Substituierten in Deutschland in Diamorphinbehandlung (1450 Personen von 80.400 Substituierten; [3]). Die Behandlung wurde zum Stichtag in nur 14 (!) Institutionen durchgeführt. Der Umfang der Gruppe der in der Standardsubstitution schwer zu Behandelnden wird unterschiedlich angegeben (zwischen 3 und 25 %, z. B. 5–10 % [9]). Dies spricht für einen Ausbau der Diamorphinbehandlung in Deutschland.

Durch die Novellierung des § 5a der BtMVV wurden die Indikationskriterien zur Diamorphinbehandlung deutlich gelockert. Die bisherigen Kriterien betonten die Schwere der Abhängigkeit (z. B. Dauer der Erkrankung) sowie die Nachrangigkeit der Diamorphinbehandlung. Tatsächlich sind Lebensalter und Dauer der Heroinabhängigkeit fragliche Anhaltspunkte für die Schwere der Heroinabhängigkeit. Bei den entsprechenden Studien gab es z. T. weniger strenge Anforderungen, z. T. aber auch strengere als in Deutschland (z. B. Mindestalter 25 Jahre in den Studien in den Niederlanden und in Kanada, siehe [5]). Der intravenöse Konsum ist hingegen ein akzeptierter Risikofaktor für schwerwiegende gesundheitliche und soziale Probleme bei Opioidabhängigkeit. Erfreulicherweise ist der Anteil der inhalativ konsumierenden Heroinabhängigen in Deutschland deutlich gestiegen, z. T. über 60 % [1].

Angesichts der psychischen und sozialen Probleme von Personen mit Heroinabhängigkeit kann eine Substitutionsbehandlung aus vielfältigen Gründen scheitern, die nur z. T. durch das verabreichte Substitutionsmittel bedingt sind. Insofern waren die nach der alten Regelung geforderten *zwei* gescheiterten Behandlungen ohnehin ein weiches Indikationskriterium für eine Diamorphinbehandlung. In der in Deutschland durchgeführten Diamorphinstudie zählte eine besondere gesundheitliche Beeinträchtigung (definiert mit dem Opiate Treatment Index) zu den Indikationskriterien [6]. Über die Erfahrung mit Substitutionsbehandlungen von insgesamt mindestens sechs Monaten dürfte die weite Mehrheit der Heroinabhängigen verfügen. Eine evidenzbasierte Begründung für die Ausweitung der Indikation durch neuere randomisierte, kontrollierte Studien ist nicht ersichtlich. Kohortenstudien berichten über positive Erfahrungen mit oralem Diamorphin in der Substitutionsbehandlung [2]. In der kontrollierten Untersuchung in den Niederlanden wurde auch inhalatives Heroin eingesetzt [7]. Der Einsatz dieser Applikationsformen erscheint sinnvoll bei Heroinabhängigen, die nicht mehr intravenös konsumieren wollen oder (bei schlechtem Venenstatus) können. Die Therapiekosten für eine Diamorphinbehandlung sind insbesondere wegen der Rahmenbedingungen (z. B. längere Öffnungszeiten mit erhöhtem Personalbedarf, behördliche Sicherheitsauflagen) deutlich höher im Vergleich zu einer Standardsubstitution – nach Einschätzung des AOK-Bundesverbands (2024) etwa 6‑mal höher. Dies spricht sozialrechtlich für die Nachrangigkeit der Diamorphinbehandlung.

## Fazit für die Praxis

- Die novellierten Kriterien (§ 5a BtMVV) helfen wenig bei der Differenzialindikation zwischen der Behandlung mit lang wirksamen Substituten und der Behandlung mit Diamorphin.
- Die organisatorischen Auflagen der Diamorphinbehandlung erfordern erhebliche Investitionen. Dies (und weniger die Indikationskriterien) begrenzt die Verfügbarkeit der Behandlung.
- Die höheren Erlöse in Großpraxen für eine Diamorphinbehandlung könnten bei unspezifischen Indikationskriterien ein ökonomischer Fehlanreiz sein.
